# Safety evaluation of curative gastrectomy for gastric cancer patients who underwent liver transplantation: a comparative study with conventional gastrectomy for gastric cancer patients

**DOI:** 10.1186/s12957-023-03028-3

**Published:** 2023-05-11

**Authors:** Chang Seok Ko, Jin Ho Jheong, Seong-A. Jeong, Byung Sik Kim, Jeong Hwan Yook, Beom Su Kim, In-Seob Lee, Chung Sik Gong, Na Young Kim, Moon-Won Yoo

**Affiliations:** 1 Department of Stomach Surgery, Asan Medical Center, University of Ulsan College of Medicine, 88, Olympic-Ro 43-Gil, Songpa-Gu, Seoul, 05505 Republic of Korea; 2 Department of Clinical Epidemiology and Biostatistics, Asan Medical Center, University of Ulsan College of Medicine, Seoul, Republic of Korea

**Keywords:** Liver transplantation, Gastrectomy, Stomach neoplasms, Complication

## Abstract

**Background:**

We aimed to examine the technical and oncological safety of curative gastrectomy for gastric cancer patients who underwent liver transplantation.

**Methods:**

In this study, we compared the surgical and oncological outcomes of two groups. The first group consisted of 32 consecutive patients who underwent curative gastrectomy for gastric cancer after liver transplantation (LT), while the other group consisted of 127 patients who underwent conventional gastrectomy (CG). In addition, a subgroup analysis was performed to evaluate the impact of the background differences and the surgical outcomes on the involvement of a specialized liver transplant surgery team.

**Results:**

The mean operative time was significantly longer in the LT group (*p* < 0.05). Furthermore, there were more frequent cases of postoperative transfusion in the LT group compared to the CG group (*p* < 0.05). However, there were no significant differences in the overall complications between the groups (25.00 vs 23.62%, *p* = 0.874). The 5-year overall survival rates of the LT and CG groups were 76.7% and 90.1%, respectively (*p* < 0.05). The results of the subgroup analysis demonstrated no statistically significant difference in various early surgical outcomes, such as time to transfusion during surgery, first flatus, time to first soft diet, postoperative complications, hospital stay after surgery, and the number of harvested lymph nodes except for operation time.

**Conclusions:**

Despite one’s medical history of undergoing LT, our study demonstrated that curative gastrectomy could be a surgically safe treatment for gastric cancer. However, further study should be conducted to identify the reason gastric cancer patients who underwent liver transplant surgery have lower overall survival rate.

## Introduction

With the increase in transplant surgeries and improved survival of transplant patients due to improvements made in immunosuppressants, de novo malignancy (DNM) in transplant patients has gained interest. DNM is a cause that negatively influences long-term survival of transplant patients [1]. According to recent research, the incidence of malignant diseases is 2–fourfold higher in transplant patients compared to the general population, and 6–15% of transplant patients develop DNM. In particular, for gastric cancer, the incidence rate is 1.6–twofold higher in organ transplant patients compared to the general population. Moreover, mortality from gastric cancer is also 1.25-fold higher in transplant patients when compared to the general population [2, 3].

According to the data collected by the Korean Network for Organ Sharing under the Ministry of Health and Welfare, more than 50,000 organ transplants have been performed in South Korea since 2000 when data collection started. In 2019, more than 5700 organ transplants were performed, approximately 26% of which were liver transplants [4].

In general, for treatment of gastric cancer in patients who received a liver transplant, curative gastrectomy through laparotomy is required, except in some cases of early gastric cancer [5]. However, technical difficulties in terms of gastrectomy and other various difficulties in preoperative management and postoperative rehabilitation are expected. For instance, since a liver transplant is performed through a large abdominal incision, significant intraabdominal adhesions are expected after surgery. Moreover, in terms of the scope of the surgery, the neo-hepatic hilum should be dissected to secure the duodenum, and lymph nodes around the common hepatic artery should be resected. In addition, collateral vessels around the portal vein may also influence safe surgery for gastric cancer treatment [6, 7]. Since patients should take immunosuppressants for life after receiving an organ transplant, postoperative immunosuppression is inevitable in transplant patients. Such immunosuppression may have a negative impact on postoperative rehabilitation [8, 9]. However, only a few case reports have been made so far regarding gastrectomy for gastric cancer in patients with liver transplants [10, 11]. Therefore, this study aimed to examine the technical and oncological safety of curative gastrectomy for gastric cancer patients who underwent liver transplantation.

## Materials and methods

### Patients

**Fig. 1:**
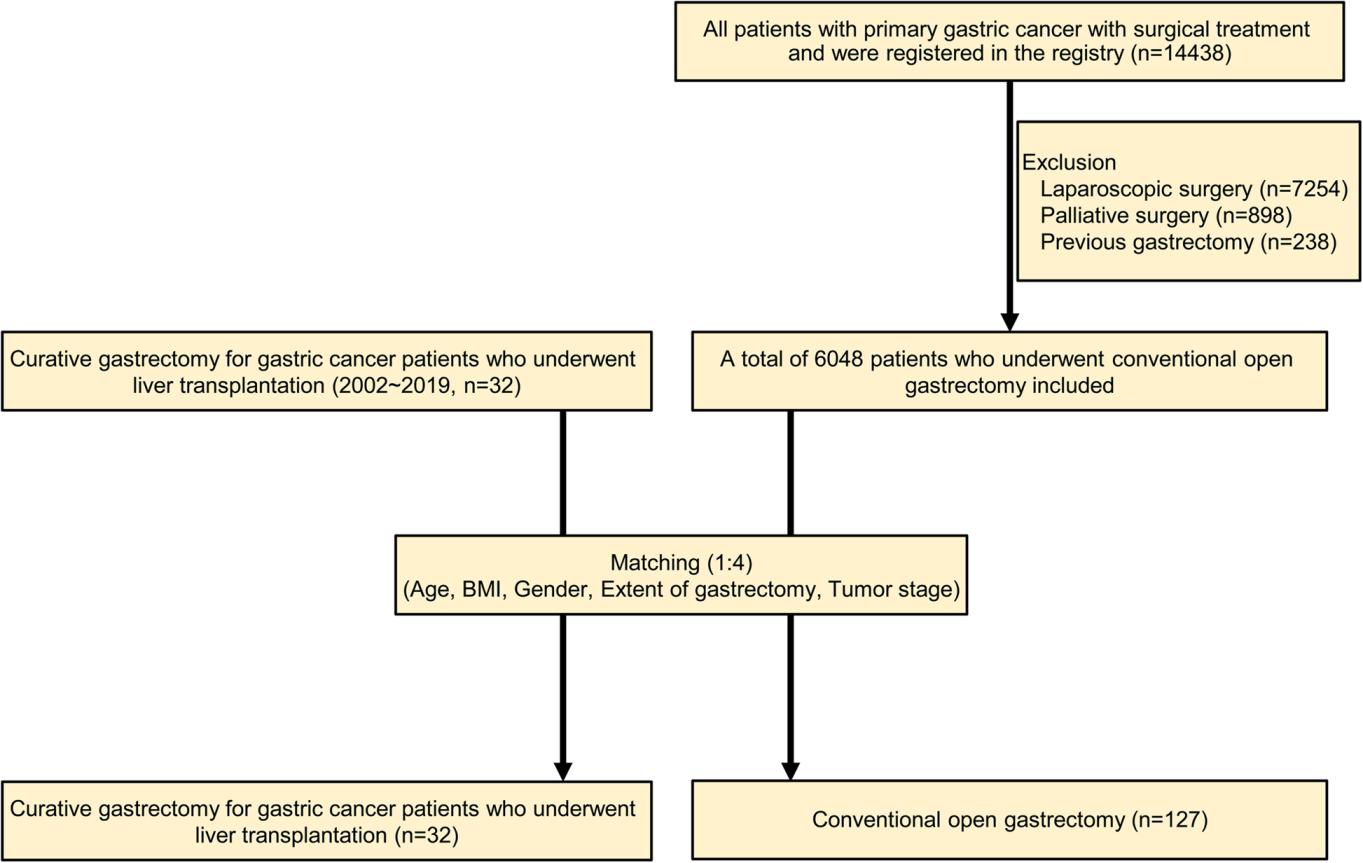
Flow chart of patient enrollment

After institutional review board approval, we compared the surgical and oncological outcomes of the two groups (2022–0794). The first group consisted of 32 consecutive patients who underwent curative gastrectomy for gastric cancer after liver transplantation (LT) between 2002 and 2019. The other group was extracted from the gastric cancer registry that consists of 14,438 patients. However, only those who received laparoscopic surgery, palliative surgery, neoadjuvant treatment, and previous gastrectomy were excluded in this study. Finally, we obtained data on 6048 patients who underwent conventional open gastrectomy (Fig. 1).

The matching was performed to compare age, gender, body mass index (BMI), extent of gastrectomy, and tumor stage. Considering the differences in preexisting underlying diseases between those who had LT and those who underwent conventional gastric cancer surgery, we have excluded factors related to comorbidity during the matching process. After matching, 32 patients who underwent a curative gastrectomy after LT (LT) and 127 who underwent conventional gastrectomy (CG) were assessed to examine the surgical and oncological outcomes.

### Data collection and outcome measurement

Patient information at the time of the liver transplant, such as age, gender, diagnoses before liver transplant, Child Pugh score, Model for End-Stage Liver Disease (MELD) score, type of liver transplant, type of biliary anastomosis, and interval between liver transplant and gastric cancer diagnosis, was gathered. Data at the time of gastric cancer surgery were collected in order to clinically evaluate surgical and oncological outcomes. This data included the patient’s age, gender, BMI, extent of gastrectomy, tumor stage according to the AJCC/UICC 7th edition [12], the presence of comorbidity, American Society of Anesthesiologist (ASA) score, combined resection, and extent of lymph node dissection. In addition, the operative time, time to first flatus, time to soft diet, intra- and postoperative transfusion, hospital stay after surgery, tumor size, number of metastatic lymph nodes, number of harvested lymph nodes, and resection margins were also collected.

The primary endpoint of this study is morbidity within 30 days after gastrectomy for safety evaluation of gastrectomy for gastric cancer patients who underwent liver transplantation compared with conventional gastrectomy. Furthermore, the complications were classified by severity using the Clavien-Dindo classification system [13]. The secondary endpoint is overall survival (OS). OS refers to the length of time between the date of surgery for gastric cancer and the date of death from any cause.

### Statistical analysis

We conducted a 1:4 case-controlled study by matching 32 curative gastrectomy cases with 127 conventional gastrectomy controls based on age, BMI, gender, type of operation, and TNM stage. A greedy algorithm was used and resulted in such pairs of subjects by randomly selecting a case and matching this to the control. After matching, cases and controls were compared for baseline characteristics of patients using a chi-squared test or Fisher’s exact test and a *t* test. To compare binary or continuous outcome variables, generalized estimating equation (GEE) was performed using identity and logit link functions, respectively. Some variables that did not follow a normal distribution were used after log transformation. Kaplan–Meier analysis was used to comparatively evaluate the recurrence-free survival (RFS) and OS rates between the groups. It was also performed using Cox models with robust standard errors that accounted for the clustering of matched pairs. The statistical software used for SAS version 9.4 (SAS Institute, Cary, NC, USA). Statistical significance was defined for all comparisons as a *p* value of less than 0.05.

## Results

The clinical information before liver transplant of the LT group is shown in Table 1. The mean age at liver transplantation was 64.06 ± 8.67 years old, and 87.50% of the patients were male. The most common pretransplant diagnosis was hepatitis B virus liver cirrhosis (71.88%). The Child Pugh score and MELD score at liver transplantation were 10.94 ± 2.20 and 21.90 ± 8.73, respectively. In terms of the details regarding the liver transplant, the dominant type was living donor liver transplant (LDLT) using the right graft (59.38%), followed by LDLT using a dual donor graft (15.63%). Approximately, 70% of patients received duct to duct anastomosis as biliary reconstruction in the liver transplantation procedure. The median interval between the liver transplantation and gastric cancer surgery was 49 months.

**Table 1:** Clinical characteristics (pre-LT)

Variable	Curative gastrectomy after LT (*n* = 32)
Age at LT	64.06 ± 8.67
Gender (M:F)	28:4
Pretransplant diagnosis	
HBV LC	23 (71.88)
HCV LC	2 (6.25)
Alcoholic LC	7 (21.88)
Other	1 (3.13)
Concurrent HCC	14 (43.75)
Child Pugh score	10.94 ± 2.20
MELD score	21.90 ± 8.73
Type of LT	
DDLT	4 (12.50)
LDLT (Rt graft)	19 (59.38)
LDLT (Lt graft)	4 (12.50)
LDLT (Dual donor)	5 (15.63)
Biliary anastomosis	
Duct to duct	22 (68.75)
Hepaticojejunostomy	10 (31.25)

Values are expressed as the mean ± SD or number (%)

*LT* liver transplant, *HBV* hepatitis B virus, *HCV* hepatitis C virus, *LC* liver cirrhosis, *HCC* hepatocellular carcinoma, *MELD* model for endstage liver disease, *DDLT* deceased donor liver transplant, *LDLT* living donor liver transplant, *SD* standard deviation

Table 2 demonstrates the baseline characteristics of the LT and CG groups after matching. As shown, the baseline characteristics, except for comorbidity and ASA scores, were balanced between the two groups. A significantly greater number of individuals from the LT group had higher comorbidity than their counterparts from the CG group (78.13 vs. 50.39%, *p* < 0.05). The number of patients with an ASA score of 3 was greater in the LT group than in the CG group (18.75 vs. 4.72%, *p* < 0.05).

**Table 2:** Baseline characteristics of the two groups after matching

Variable	Matching set (1:4) (*n* = 159)		*P* value
	Curative gastrectomy after LT (*n* = 32)	Conventional gastrectomy (*n* = 127)	
Age	61.58 ± 8.33	61.22 ± 7.53	0.822
Gender			> 0.999
Male	28 (87.5)	111 (87.4)	
Female	4 (12.5)	16 (12.6)	
BMI	23.06 ± 3.27	22.55 ± 2.87	0.420
Extent of gastrectomy			0.980
Distal	23 (71.88)	91 (71.65)	
Total	9 (28.13)	36 (28.35)	
Tumor stage			> 0.999
I	23 (71.88)	92 (72.44)	
II	5 (15.63)	19 (14.96)	
III	4 (12.5)	16 (12.6)	
Comorbidity			0.005
Yes	25 (78.13)	64 (50.39)	
No	7 (21.88)	63 (49.61)	
ASA score			0.016
1,2	26 (81.25)	121 (95.28)	
3	6 (18.75)	6 (4.72)	
Combined resection			0.262
Yes	4 (12.5)	8 (6.3)	
No	28 (87.5)	119 (93.7)	
LN dissection			0.111
Less than D2	22 (68.75)	65 (51.18)	
D2	10 (31.25)	62 (48.82)	
Adjuvant chemotherapy			0.164
Yes	5 (15.63)	35 (27.56)	
No	27 (84.38)	92 (72.44)	

Values are expressed as the mean ± SD or number (%)

*LT* liver transplant, *BMI* body mass index, *ASA* American Society of Anesthesiologists Physical Status Classification, *LN* lymph node, *SD* standard deviation

The early surgical outcomes and pathologic data are presented in Table 3. The mean operative time was significantly longer in the LT group (*p* < 0.05). Furthermore, the patients in the LT group required postoperative transfusion more frequently than those in the CG group (25.00 vs. 7.87%, *p* < 0.05). The hospital stay was significantly longer in the LT group. The number of harvested lymph nodes was significantly fewer in the LT group (18.91 ± 11.89 vs. 32.49 ± 14.81, *p* < 0.05). However, other surgical outcomes and pathologic data such as time to first flatus, time to soft diet, intraoperative transfusion, readmission rate, tumor size, tumor stage, nodal stage, and metastatic lymph nodes were not significantly different between the groups.

**Table 3:** Early surgical outcomes and pathologic data of the two groups after matching

Variable	Matching set (1:4) (*n* = 159)		*P* value
	Curative gastrectomy after LT (*n* = 32)	Conventional gastrectomy (*n* = 127)	
Operative time^a^	5.27 ± 0.32	4.89 ± 0.25	< 0.0001
Time to first flatus (days)	3.94 ± 1.13	3.86 ± 0.96	0.738
Time to soft diet^a^	1.70 ± 0.37	1.58 ± 0.31	0.070
Intraoperative transfusion (*n*)	3 (9.38)	0 (0.00)	NA
Postoperative transfusion (*n*)	8 (25.00)	10 (7.87)	0.015
Hospital day^a^	2.34 ± 0.39	2.18 ± 0.31	0.019
Readmission (*n*)	1 (3.13)	3 (2.36)	0.790
Tumor size (cm)	3.71 ± 2.30	4.16 ± 2.47	0.310
T stage			0.144
1	17 (53.13)	77 (60.63)	
2	7 (21.88)	26 (20.47)	
3	4 (12.50)	15 (11.81)	
4	4 (12.50)	9 (7.09)	
*N* stage			0.210
0	26 (81.25)	93 (73.23)	
1	3 (9.38)	12 (9.45)	
2	2 (6.25)	12 (9.45)	
3	1 (3.13)	10 (7.87)	
Metastatic LN (*n*)	1.87 ± 5.30	0.91 ± 3.13	NA
Harvested LN (*n*)	18.91 ± 11.89	32.49 ± 14.81	< 0.0001
PRM (cm)	4.48 ± 2.96	5.01 ± 2.87	0.396
DRM^a^	1.45 ± 0.91	1.44 ± 0.94	0.916

Values are expressed as the mean ± SD or number (%)

*LT* liver transplant, *LN* lymph node, *PRM* proximal resection margin, *DRM* distal resection margin, *SD* standard deviation

^a^analysis after log-transformation

Table 4 presents the details of postoperative complications for the two groups. As shown in the table, there were no significant differences in overall complications between the groups (25.00 vs. 23.62%, *p* = 0.874). In addition, the results displayed no significant differences in ≥ 3 CDC complications, anastomotic complications, postoperative bleeding, fluid collection, and wound complications between the two groups (*p* > 0.05). There were no differences between the 30 and 90 days morbidity, and there was no mortality within 90 days after surgery in both groups.

**Table 4:** Postoperative complications of the two groups after matching

Variable	Matching set (1:4) (*n* = 159)		*P* value
	Curative gastrectomy after LT (*n* = 32)	Conventional gastrectomy (*n* = 127)	
Overall complication	8 (25.00)	30 (23.62)	0.874
CDC ≥ 3 complication	1 (3.13)	9 (7.09)	0.401
Anastomotic complication	0 (0.00)	4 (3.15)	0.998
Postop bleeding	1 (3.13)	6 (4.72)	0.707
Fluid collection	1 (3.13)	3 (2.36)	0.803
Wound complication	3 (9.38)	8 (6.3)	0.54

Values are expressed as the mean ± SD or number (%)

*LT* liver transplant, *CDC* Clavien–Dindo classification, *SD* standard deviation

In order to evaluate the impact of the involvement of a specialized liver transplant surgery team on the surgical outcomes of gastrectomy, we divided 32 liver transplant patients into two groups: one with patients whose surgery was performed by a stomach surgery team alone (hereafter, ST) and the other with those whose surgery involved a specialized liver transplant surgery team as well as a stomach surgery team (hereafter, LST). There were no statistically significant differences in the baseline characteristics of the two groups. Table 5 compares the early surgical outcomes of liver transplant patients between the ST and LST groups. The mean operative times were 160.90 ± 43.15 and 244.00 ± 57.01 min for the ST group and the LST group, respectively (*p* < 0.0001). However, no significant differences were found between the two groups in terms of other early surgical outcomes, such as time to transfusion during surgery, first flatus, time to first soft diet, postoperative complications, hospital stay after surgery, and the number of harvested lymph nodes.

**Table 5:** Background characteristics and surgical outcomes of the patients who underwent curative gastrectomy after liver transplantation

Variable	Stomach surgery team alone (*n* = 15)	Involved a specialized liver transplant surgery team (*n* = 17)	*P* value
Type of LT			0.893
DDLT	1 (6.67)	3 (17.65)	
LDLT: Right lobe	9 (60.00)	10 (58.82)	
LDLT: Left lobe	2 (13.33)	2 (11.76)	
LDLT: Dual donor	3 (20.00)	2 (11.76)	
Biliary anastomosis			> 0.999
Duct to duct	10 (66.67)	12 (70.59)	
Hepaticojejunostomy	5 (33.33)	5 (29.41)	
Operative time (min)	160.90 ± 43.15	244.00 ± 57.01	< 0.0001
Intraoperative transfusion (*n*)	1 (6.67)	2 (11.76)	0.626
Time to first flatus (days)	4.00 ± 1.25	3.88 ± 1.05	0.775
Time to soft diet (days)	5.47 ± 1.77	6.12 ± 1.80	0.311
Overall complication	4 (26.67)	4 (23.53)	0.838
CDC ≥ 3 complication	0 (0.00)	1 (5.88)	NA
Anastomotic complication	0 (0.00)	0 (0.00)	NA
Hospital day (*n*)	10.60 ± 4.94	9.90 ± 7.27	0.493
Harvested LN (*n*)	18.60 ± 13.24	19.17 ± 10.97	0.894

Values are expressed as the mean ± SD or number (%)

*LT* liver transplant, *DDLT* deceased donor liver transplant, *LDLT* living donor liver transplant, *CDC* Clavien–Dindo classification, *LN* lymph node, *SD* standard deviation

**Fig. 2:**
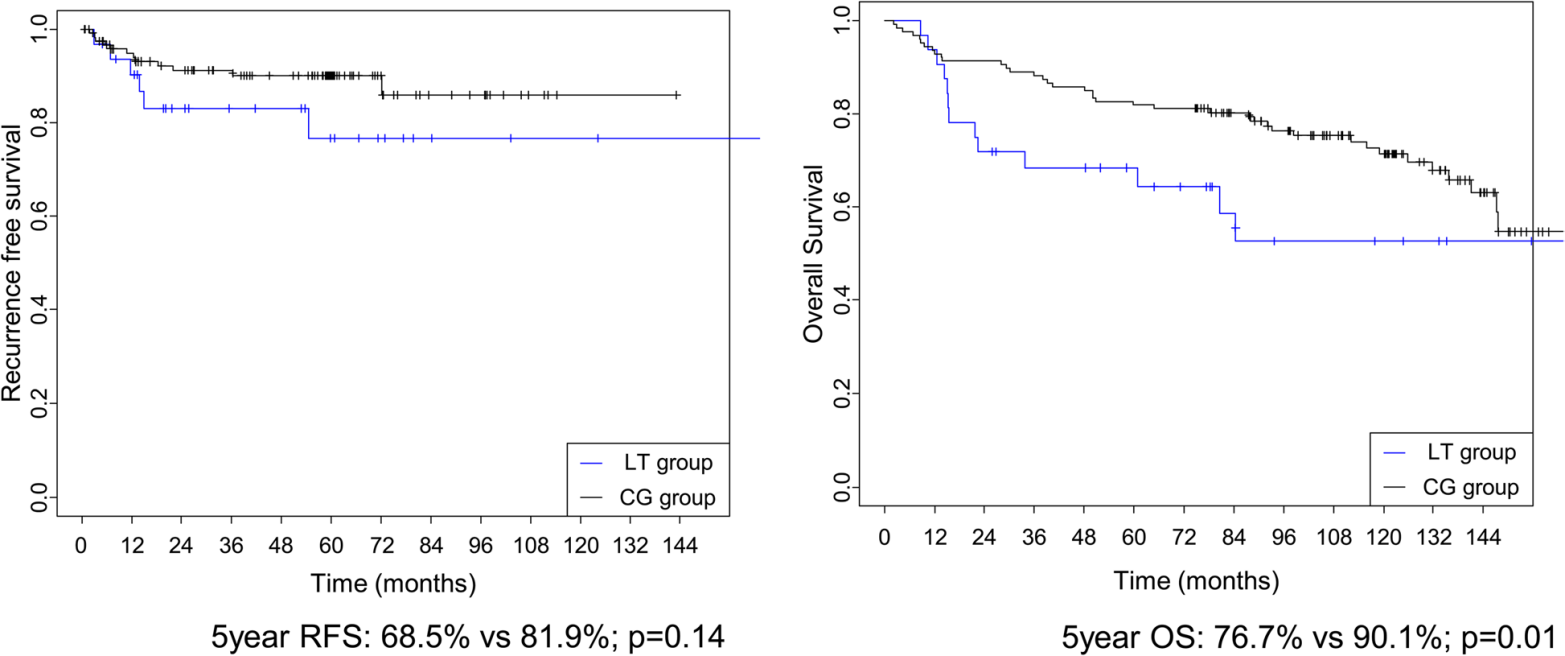
Recurrence free survival and overall survival after matching

Figure 2 shows the OS and RFS by group. The 5-year OS rates of the LT and CG group were 76.7% and 90.1%, respectively. The difference between the groups was statistically significant (*p* < 0.05). Although the 5-year RFS rate of the LT group was lower than that of the CG group (68.5 vs 81.9%), the difference was found to be non-significant (*p* > 0.05) and there was no difference in the recurrence pattern between the groups. Additionally, we analyzed the respective interactions of OS and RFS with the tumor stages by group, and the results are shown in Table 6. It shows that there were no significant interaction effects in both groups (*p* > 0.05).

**Table 6:** Overall survival and recurrence-free survival of the two groups after matching

	Group	3 years			5 years			Interaction *p* with stage
		Estimate	95%CI		Estimate	95%CI		
RFS	LT group	0.685	0.54	0.868	0.685	0.54	0.868	0.7215
	CG group	0.882	0.828	0.94	0.819	0.755	0.889	
OS	LT group	0.831	0.705	0.978	0.767	0.611	0.962	0.5615
	CG group	0.912	0.861	0.966	0.901	0.847	0.959	

*CI* confidence interval, *RFS* recurrence-free survival, *OS* overall survival

## Discussion

Previous studies mostly analyzed the incidence of gastric cancer after a liver transplant and argued for the need for regular endoscopic screening to detect gastric cancer early [5, 14]. Moreover, studies on the surgical treatment of gastric cancer after a liver transplant were mostly case reports [6, 7, 10, 11]. The present study is the first to comparatively analyze the postoperative and oncological outcomes of gastric cancer patients with liver transplants who received a gastrectomy. This study confirmed that curative gastrectomy is possible in the surgical treatment of gastric cancer in liver transplant patients.

Although differences are expected between studies, the postoperative complication rate after gastrectomy for gastric cancer has been reported to range between 10 and 30%. The major complication rate with CDC grade 3 or above is approximately a 10% [15, 16]. In the present study, the group who received gastrectomy following a liver transplant had a 20% complication rate and a 1% major complication rate. Moreover, postoperative mortality in 30 days and 90 days was 0%. This suggests that the surgery was safe even when compared to other studies on conventional gastrectomy.

The number of harvested lymph nodes was significantly fewer in the LT group in this study. We believe that the reason the LT group had significantly fewer harvested lymph nodes is that the lymphadenectomy was already performed around the hepatoduodenal ligament in the liver transplant surgery.

As shown, with the help of an experienced liver transplant surgery team, the gastrectomy of patients who previously received liver transplant had comparably favorable outcomes with those who did not have a liver transplant specialized team on their surgery. The decision around the involvement of the specialized liver transplant surgery team was made practically by the gastric cancer surgeon based on the severity and complexity of the case. Nagata et al. reported that unusual variations of vessels require attention while preforming gastrectomy on liver transplant patients and that information on the course of blood vessels should be obtained preoperatively to prevent serious problems [6]. The liver transplant team helped with adhesiolysis around the neo-hepatic hilum, and with the identification of the hepatic artery, portal vein, and bile duct. Moreover, in order to secure the distal margin, the transplant team assisted with adequate exposure of the first portion of the duodenum. We believe such organic cooperation system during surgery and preoperative anatomical understanding of the liver transplant surgery had led to a safe and favorable outcome of gastrectomy in patients who previously received liver transplant.

Lee et al. showed that laparoscopic gastrectomy is possible in liver transplant patients [10]. However, in many liver transplant patients in whom strong adhesions of hepatic hilum require dissection, laparoscopic surgery may not be adequate for careful dissection. Moreover, iatrogenic injury from energy devices also requires caution. Kun Yang claimed that open surgery should be the optimal procedure for adhesions and oncologic safety. Kun Yang also mentioned that dissection of 5, 8, and 12 lymph nodes should be performed very carefully while paying attention to reconstructed vessels and bile ducts in order to avoid iatrogenic injuries [7]. Also, in the present study, gastrectomy was performed through laparotomy, which is relatively safer than experimental laparoscopy, in all patients.

According to previous studies, immunosuppression, such as that from long-term use of steroids, may be a factor associated with leakage of intestinal anastomosis. Moreover, immunosuppression prolongs hospital stays by causing surgical site infections and other various medical problems and increases reoperation, readmission, and mortality rates [8, 9]. In particular, since liver transplant patients should take immunosuppressants for life to prevent graft rejection, this is expected to have a negative impact on postoperative complete healing of the anastomotic site. Notwithstanding the small sample size, there was no anastomotic site leakage observed in the present study. This is likely because the surgeons had ample experience in gastrectomy and performed careful reinforcement sutures around the anastomotic site. Moreover, the number and size of meals were reduced postoperatively, and the patients were instructed to start with a liquid diet initially. They were then slowly transitioned to a solid diet after resolution of the paralytic ileus. These efforts to lessen the pressure on anastomotic sites would have also prevented complications at the sites.

Previous studies reported that the 5-year OS rate after liver transplant ranged from 68 to 87% [17, 18]. This is significantly lower than that of the general population. To be specific, the difference in the median survival time between the general population and the patients with liver transplant was approximately seven life years [19]. Such vulnerabilities of those who underwent liver transplant are due to immune deficiency, DNM, cardiovascular disease, and previous liver disease after a liver transplant [18]. The findings of our study are consistent with previous studies, showing lower survival rates in the LT group compared to the CG group.

The present study has a few limitations. First, this study was a single-center, retrospective study. Second, the sample size of the LT group was relatively small. Therefore, future studies should include larger patient groups.

## Conclusion

In conclusion, this study showed that gastrectomy can be safely performed in liver transplant patients. However, further study should be conducted to identify the reason gastric cancer patients who underwent liver transplant surgery have lower overall survival rate compared to those who did not undergo liver transplant surgery.

## Data Availability

The data that was analyzed in this study are available from the corresponding author upon acceptable request.
